# Predisposing factors for poor outcomes after intense pulsed light treatment for dry eye disease: A retrospective case-control study

**DOI:** 10.7150/ijms.101341

**Published:** 2024-11-04

**Authors:** Chia-Yi Lee, Shun-Fa Yang, Hung-Chi Chen, Chao-Kai Chang

**Affiliations:** 1 Institute of Medicine, Chung Shan Medical University, Taichung, Taiwan.; 2 Nobel Eye Institute, Taipei, Taiwan.; 3 Department of Ophthalmology, Jen-Ai Hospital Dali Branch, Taichung, Taiwan.; 4 Department of Medical Research, Chung Shan Medical University Hospital, Taichung, Taiwan.; 5 Department of Ophthalmology, Chang Gung Memorial Hospital, Linkou, Taiwan.; 6 Center for Tissue Engineering, Chang Gung Memorial Hospital, Linkou, Taiwan.; 7 Department of Medicine, Chang Gung University College of Medicine, Taoyuan, Taiwan.; 8 Department of Optometry, Da-Yeh University, Chunghua, Taiwan.

**Keywords:** dry eye disease, intense pulse light, tear break-up time, refractive surgery, meibomian gland

## Abstract

**Purpose:** To evaluate the potential risk factors for poor dry eye disease (DED) outcomes after intense pulse light (IPL) treatment.

**Methods:** A retrospective case-control study was conducted, and patients who received IPL were enrolled. A total of 63 eyes were included in the present study after exclusion and were divided into a fair outcome group and a poor outcome group according to posttreatment improvements in DED-related signs and symptoms. The primary outcomes are the pretreatment parameters between the two groups. The Mann‒Whitney U test and generalized linear model were adopted to analyze the differences in pretreatment indices between the two groups.

**Results:** Both the fluorescein stain results and the Schirmer II test results after IPL treatment were significantly better than those before IPL treatment (both P < 0.05). Nevertheless, the overall DED-related symptoms did not significantly improve after IPL treatment (P = 0.834). In terms of indicators of poor outcomes after IPL treatment, the rates of advanced age, female sex, previous refractive surgery, lower pretreatment noninvasive tear break-up time (NITBUT) and greater meibomian gland loss were significantly greater in the poor outcome group (all P < 0.05). Female sex and previous refractive surgery were associated with less improvement in DED-related symptoms (all P < 0.05), whereas advanced age, a lower pretreatment NITBUT and a higher meibomian gland loss rate were related to poor DED sign improvement (all P < 0.05).

**Conclusion:** The major limitations are the retrospective design, small study population, and absence of detailed posttreatment exams. In conclusion**,** old age, female sex, previous refractive surgery, a lower NITBUT, and a higher meibomian gland loss rate are associated with worse outcomes after IPL treatment.

## Introduction

Dry eye disease (DED) is a widespread disease that affects most of the population and can be categorized into evaporative excess, aqueous deficiency, mucin deficiency and mixed types 1. According to a previous epidemiological study, the prevalence of DED in the Asian population is approximately 20% 2. The common symptoms of DED include dryness, grittiness, discharge sensation, foreign body sensation and photophobia 3. Advanced DED may contribute to visual disturbance and decreased vision 4, and neurotrophic keratitis can also develop in those with severe DED 5.

Several medical and surgical methods have been proposed for the management of DED 6. Artificial tears have been widely used for general DED and postoperative DED previously 7, 8. The ingredients of artificial tears are various 9, and hyaluronic acid-containing artificial tears have gained popularity in recent years because of their anti-inflammatory and regenerative functions, which can retard DED symptoms and signs 10-12. On the other hand, steroids have been applied in DED and are fairly effective; the combined use of steroids and artificial tears can significantly reduce the severity of DED, and the safety of this treatment is also acceptable 13. In refractory DED patients, surgical management, including amnion membrane transplantation, may be considered to preserve the damaged ocular surface 14.

Intense pulsed light (IPL) therapy was introduced as a DED treatment in early 2000 15. The IPL refers to a nonlaser high-intensity light resource with a large wavelength range from approximately 500-1200 nm 16. By using the selected cutoff filter, the IPL device can emit the optimal wavelength to specific epidermal and dermal structures at a particular depth 17, which is referred to as the selective photothermolysis effect 16. In the ocular area, the known mechanisms of IPL for DED reduction include Demodex eradication, thrombosis of abnormal vessels, photobiomodulation, and meibum substance fluidification 18. Additionally, IPL can alleviate the inflammatory response in DED by increasing the levels of anti‑inflammatory factors or retarding the levels of proinflammatory factors 19. In a previous review article, the application of IPL effectively reduced DED-related symptom scores based on the ocular surface disease index and the standard patient evaluation of eye dryness 20. For objective signs, IPL has good effectiveness on meibum quality and invasive and noninvasive tear break-up times in randomized studies 16, 17. In addition, the heating effect of IPL therapy results in good DED retardation efficiency, especially for those with evaporative excess DED 21, 22. Additionally, IPL therapy is more effective for evaporative-excess DED than artificial tear therapy is 23. Nevertheless, few studies have evaluated the predisposing factors for poor therapeutic outcomes of IPL therapy. Since DED-related factors such as sex can influence the treatment outcome of artificial tears 24, certain parameters may also affect the efficacy of IPL therapy.

Consequently, the objective of the current study was to survey the potential risk factors for the poor prognosis of DED patients after IPL therapy. DED-related signs and symptom improvements were included and analyzed in the current study. To our knowledge, the pretreatment factors that cause poor DED improvement after IPL treatment were reported for the first time in the present study, and we also identified the predictors of DED-related signs and symptom improvement after IPL treatment separately. These findings may help physicians judge whether to add additional DED therapy to patients scheduled for IPL to improve DED.

## Materials and methods

### Ethics declaration

This study adhered to the Declaration of Helsinki in 1964 and its accompanying amendments. Furthermore, the current study was approved by the Institutional Review Board of National Changhua University of Education (project code: NCUEREC-113-056). The essence of written informed consent was postponed by the Institutional Review Board of National Changhua University of Education because of the retrospective nature of the current study.

### Participant selection

A retrospective case-control study was performed at the Nobel Eye Institute, a group that owns multiple clinics in the Taiwan region. The patients were included in the current study if they met the following criteria: (1) age from 20 to 100 years, (2) received a diagnosis of DED at the Nobel Eye Institute, (3) received an automatic ocular surface analyzer at the Nobel Eye Institute, (4) received IPL treatment at least three times at the Nobel Eye Institute, and (5) were followed up at the Nobel Eye Institute for at least six months. On the other hand, the following exclusion criteria were adopted to exclude patients with impaired ocular surfaces: (1) previous central corneal opacity, (2) previous corneal ulcer episode, (3) previous corneal erosion, (4) previous deep corneal infiltration, (5) previous corneal perforation, (6) previous chemical burn that involved the cornea, and (7) previous cicatricial conjunctivitis. Only the right eye of each DED patient was included in the current study. A total of 75 patients who received IPL treatment were screened, and 3 of them were excluded because of central corneal opacity; 3 of them were excluded because of previous corneal ulcers; 2 of them were excluded because of previous corneal erosion; and 1 patient each was excluded because of previous deep corneal infiltration, previous corneal perforation, previous chemical burn and previous cicatricial conjunctivitis. After the selection process, 63 eyes were included in the current study.

### Intense pulse light treatment

All the IPL treatments were performed by one DED specialist (H.-C.C.) and one IPL device (M22 Optima IPL, Lumenis Be Ltd., Yokneam Industrial Park, Yokneam, Israel), and all the eyes received the same IPL treatment protocol. Local anesthesia gel was applied to the upper nasal area, upper eyelid and lower eyelid, and the patients rested for three minutes. A protective shield was placed on the cornea before the start of IPL treatment. Then, the 8x15 mm square probe of the IPL was applied to the right upper eyelid areaand the IPL was emitted 10 times with an energy of 15 J/cm^2^, and the same energy was applied to the right lower eyelid for another 10 strikes. The duration of the treatment sessions was 6 milliseconds, and the interval between sessions was 50 milliseconds. After the complement of the right eyelid treatment, the left eyelid received the same treatment protocol, and the IPL treatments for both eyelids were repeated one additional time. Finally, the patient was brought to the slit-lamp biomicroscope, and eyelid extrusion was executed to remove the meibomian substances. Artificial tears, topical fluorometholone and carbomer ointment were applied after the entire IPL treatment.

### Dry eye examinations

All the patients in the current study received the same pretreatment and post-treatment exams. A history of ocular diseases or ophthalmic surgery was obtained via medical records. The pretreatment exams include UDVA measurements, cyclopegic refraction of both sphere and cylinder powers by an autorefractor (KR-8900, Topcon, Itabashi-ku, Tokyo, Japan) and intraocular pressure (IOP) by pneumatic tonometry (NT-530, NIDEK, Gamagori, Aichi, Japan). For the DED-related parameters, the noninvasive tear break-up time (NITBUT), lipid thickness, tear meniscus height (TMH), eyelid closure rate, and meibomian gland loss rate were obtained via an automatic ocular surface analyzer (Sbm Device, SBM Sistemi, Strada Torino, Orbassano, Italy). In addition, the Schirmer II test and ocular surface staining by fluorescein were also performed in all patients. The grades of ocular surface staining were based on the Oxford Scheme. For subjective DED symptoms, dryness, itch, foreign body sensation, burning sensation, gritty, soreness, discharge, photophobia, and redness were recorded in medical documents. The routine posttreatment exams of IPL treatment also involve the UDVA, IOP, and manifest refractions by the same devices. In addition, fluorescein staining, the Schirmer II test and subjective DED symptoms were surveyed after IPL treatment. The DED-related exams before and 6 months after IPL treatment were recorded.

### Statistical analysis

SPSS version 20.0 (SPSS Inc., Chicago, Illinois, USA) was used for the statistical analysis in the current study. The statistical power of the current study was 0.75, with a 0.05 alpha value and a medium effect size, which was generated via G∗power version 3.1.9.2 (Heinrich Heine Universität at Düsseldorf, Germany). The Shapiro-Wilk test was used to check the normality of the data, and the results demonstrated nonnormal distributions of all the data in the current study (all P < 0.05). A descriptive analysis of the basic characteristics of the whole study group was performed. Then, the Wilcoxon signed rank test and chi-square test were used to compare pretreatment and posttreatment parameters. For the possible risk factors for poor outcomes, the patients were divided into a fair outcome group and a poor outcome group, in which the inclusion criteria for the poor outcome group included (1) no relief of any DED-related symptoms three months after IPL treatment, (2) improvement in the Schirmer II test score of less than 3 mm, and (3) improvement in the fluorescein stain score of less than 1 grade. In the next step, the Mann‒Whitney U test and chi‒square test were used to compare the pretreatment parameters between the two groups. In addition, the whole study group was also divided according to (1) any symptom improvement and (2) any sign improvement in the Schirmer II test or fluorescein stain, and a generalized linear model was used to compare the pretreatment parameters between the different groups. Statistical significance was defined as P < 0.05 in the present study, and a P-value less than 0.001 was considered P < 0.001.

## Results

The baseline characteristics are presented in Table 1. The mean age was 45.30 years, and there were 15 males and 48 females in the study population. The per-treatment UDVA was 0.25, and the refractive status via SE was -2.73 diopters (D) in the study population. The pretreatment DED parameters and DED-related symptoms are presented in Table 1.

After the entire treatment course, UDVA did not significantly differ between the pretreatment and posttreatment statuses (P = 0.074). In addition, the IOP and refractive status did not significantly change after IPL treatment (all P > 0.05). For the DED parameters, both the fluorescein stain and Schirmer II test results demonstrated significant improvements compared with the pretreatment status (both P < 0.05) (Table 2). Nevertheless, the overall DED-related symptoms did not significantly improve after IPL treatment (P = 0.834) (Table 2).

Concerning indicators of poor outcomes after IPL treatment, the rates of advanced age, female sex, previous refractive surgery, lower pretreatment NITBUT and greater meibomian gland loss were significantly greater in the poor outcome group (all P < 0.05) (Table 3). For indicators for poor DED symptom improvement, female sex and previous refractive surgery were associated with improved DED-related symptoms (all P < 0.05) (Table 4). On the other hand, older age, lower pretreatment NITBUT and higher meibomian gland loss rates were observed in the population with poor DED sign improvement (all P < 0.05) (Table 5).

## Discussion

In the present study, the posttreatment DED signs significantly improved compared with the pretreatment signs. Moreover, advanced age, female sex, previous refractive surgery, a lower pretreatment NITBUT and a higher meibomian gland loss rate were correlated with poor outcomes after IPL treatment. On the other hand, the indicators for poor DED symptom improvement and poor DED symptom improvement were different.

Several pathways contribute to the development of DED previously 10, 25. The loss of ocular surface homeostasis and reduced tear film stability are the major factors associated with the occurrence of DED 1. Reduced tear film stability damages the corneal epithelium and triggers the expression of several cytokines, and these two factors increase tear film osmolarity, which further contributes to an unstable tear film 26. Consequently, a vicious cycle of DED occurs 26. Inflammatory biomarkers are crucial in the process of DED, and interleukin levels increase in individuals with DED 27. Additionally, the cytokines tumor necrosis factor and interferon are also increased during DED 28. On the other hand, oxidative stress is another major pathway for DED development 29. In a previous study, high oxidative stress harmed the DNA structure and caused lipid peroxidation, both of which led to consecutive DED 30. In another study, the antioxidant levels decreased markedly in individuals who underwent keratorefractive surgery and were diagnosed with preexisting DED 31. In addition, ocular surface damage can also cause DED occurrence 32, in which impaired corneal epithelium was associated with poor tear film stability and more subjective symptoms in previous studies, and these two conditions are components of DED 5, 10. In addition, the goblet cell insult caused by chemical burn and autoimmune disease could impair mucin secretion and cause mucin deficiency in DED 25. The IPL resolves DED mainly through thermal effects and the subsequent retardation of meibomian gland dysfunction 17, and DED conditions can be improved after the excretion of meibum substances 17. Accordingly, the main mechanism of IPL in DED treatment is the reduction in evaporation. However, some predisposing factors that influence other DED pathways or lead to excessive evaporation may reduce IPL treatment effectiveness. The concept was supported by the current study findings, at least to some degree.

Compared with patients with fair outcomes, patients with poor outcomes after IPL treatment presented some demographic and ocular parameters. In previous studies, patients with advanced age may need additional management other than artificial tears for DED treatment than younger individuals do 33. In addition, individuals with autoimmune diseases such as Sjögren's syndrome also experienced less improvement in DED after artificial treatment than those without such comorbidities did 34. Nevertheless, research evaluating the factors that contribute to poor outcomes after IPL treatment is lacking. To our knowledge, our findings may provide preliminary evidence of the predisposing factors for poor outcomes after IPL treatment. Moreover, all the individuals received IPL treatment from one DED specialist; thus, the influence of the physician technique on the therapeutic outcome of IPL could be eliminated. In addition, all the patients received three IPL treatments at our institution within three months; thus, the frequencies and durations of IPL treatments for all the patients were universal. Consequently, old age, female sex, previous refractive surgery, a lower pretreatment NITBUT and a higher meibomian gland loss rate could be independent risk factors for poor outcomes after IPL treatment. Older age, female sex and previous refractive surgery are known risk factors for DED development 1, and these factors may diminish the effectiveness of any DED management, including IPL treatment. A low NITBUT is correlated with poor tear film stability, which is a fundamental factor for DED development 35, and meibomian gland extinction is related to a shortage of lipid components in the tear film 25. The results of the present study may imply that IPL is less effective in individuals with worse lipid secretion and generally severe DED.

In the analysis stratified by DED symptoms, female sex and previous refractive surgery were related to lower degrees of DED-related symptom improvement after IPL treatment. Few studies have demonstrated this correlation. Compared with male sex, female sex is correlated with greater severity of DED 36, and the overall DED-related symptoms of females are greater than those of males 37. Thus, the female population may have experienced more severe DED-related symptoms than the male population did in the current study, and those symptoms did not improve as much as those experienced by the male population after IPL treatment. Refractive surgery can cause ocular surface damage 38, and DED development is not uncommon after refractive surgery, including laser *in situ* keratomileusis 39. Even a period after refractive surgery, the sensitivity of the ocular surface nerve plexus may increase because of stimulation from the early postoperative period of refractive surgery 40. We speculate that the general sensitivity of the ocular surface in those who underwent previous refractive surgeries was elevated; thus, the decrease in symptoms after IPL treatment was lower than that in the population without previous refractive surgeries. On the other hand, advanced age, a lower pretreatment NITBUT score and a higher percentage of meibomian gland loss were associated with a lower degree of DED-related sign improvement. Old age was associated with lower tear secretion in a previous study, and the inflammatory reaction of the tear film increased in elderly individuals 33, which could lead to frequent corneal surface injury. The low NITBUT and high meibomian gland loss rate could contribute to the high inflammatory status of the ocular surface 5, 25, 41; thus, ocular surface healing from DED after any treatment could be slower under such conditions. Consequently, IPL may not be highly efficient for DED-related sign reduction because of these factors.

For the efficiency of IPL treatment in the present study, the results of the Schirmer test improved by approximately 40% after three IPL treatments. In addition, the improvements in ocular surface strain were also significant compared with those in the pretreatment conditions. In a previous study, the results of the Schirmer test after IPL treatment were also significantly improved compared with those before IPL treatment 42, whereas another study reported no improvement in the Schirmer test results after IPL treatment 43. The Schirmer test results of the current study may be compatible with those of previous studies. In addition, ocular surface stain significantly improved after IPL treatment in the present study, in which only 27% of patients maintained an advanced ocular surface stain status after IPL treatment. In an earlier publication, the corneal staining score was also reduced after IPL treatment 43, and the results of the present study were similar to those of a previous study. On the other hand, DED-related symptoms did not decrease significantly after IPL treatment, and many patients still presented with multiple DED symptoms. DED-related symptoms were significantly reduced after IPL treatment in a preceding article 22. In general, the treatment effect of the IPL in the current study may not have been inferior to that of previous experiences, although a lower degree of symptom recovery was found.

There are several limitations in the current study. First, the retrospective design of the current study reduces the homogeneity of the study populations compared with prospective research. Second, the total number of eyes included in the present study was relatively small, with only 63 eyes from 63 patients included. Although the statistical power might be acceptable, the low-case numbers may have contributed to a statistical bias. In addition, we only performed automatic ocular surface analyzer examinations before IPL treatment; thus, several crucial parameters, including the posttreatment NITBUT and posttreatment TMH, could not be assessed. Additionally, we did not use a structural questionnaire such as the ocular surface disease index questionnaire or the dry eye-related quality of life score to evaluate DED-related symptoms in our patients, and the accuracy of DED-related symptom evaluation could be reduced. Finally, all the patients enrolled in the current study were Han Taiwanese; thus, the external validity of the current study is limited.

In conclusion, advanced age, female sex, low tear film stability, high ocular surface strain and poor meibomian gland conditions correlated with poor therapeutic outcomes after IPL treatment. Furthermore, the factors associated with poor DED signs and DED symptom recovery after IPL differ. As a consequence, additional DED treatments for patients with the above predisposing factors who are scheduled for IPL treatment may be warranted to improve treatment outcomes. Further large-scale prospective research investigating whether systemic inflammatory disease affects the effectiveness of IPL treatment is needed.

## Figures and Tables

**Table 1:** Study population baseline features

Feature	Study group (N: 63)
Age (years, mean ± SD)	45.30±14.28
Sex (male: female)	15:48
Ocular disease	
Retinal disease	3
Glaucoma	1
Uveitis	1
Other	0
Ocular surgery	
Retinal surgery	1
Refractive surgery	5
Other	0
UDVA (LogMAR)	0.25±0.16
Cycloplegia refraction (D)	
Sphere	-2.26±1.87
Cylinder	-0.94±0.58
SE	-2.73±2.04
IOP	16.59±3.77
NITBUT	7.03±3.43
Eyelid closure rate	94.54±9.53
Lipid thickness	
Grade A-C	26
Grade D-E	37
TMH	0.31±0.18
Meibomian gland loss rate	29.25±17.91
Schirmer II test	10.38±3.05
Ocular surface stain	
Grade 0-3	45
Grade 4-6	18
DED-related syndrome	
1	16
2	25
≥ 3	22

D: diopter, DED: dry eye disease, N: number, SD: standard deviation, SE: spherical equivalent, NITBUT: non-invasive tear break-up time, TMH: tear meniscus height, UDVA: uncorrected distance visual acuity

**Table 2:** Ophthalmic parameters after intense pulsed light treatment compared to pre-treatment status

Outcome	Pre-treatment	Post-treatment	P
UDVA	0.25±0.16	0.20±0.14	0.074
SE (D)	-2.73±2.04	-2.80±2.02	0.853
IOP	16.59±3.77	17.38±4.21	0.248
Schirmer II test	10.38±3.05	14.56±2.78	<0.001*
Ocular surface stain			0.005*
Grade 0-3	45	58	
Grade 4-6	18	5	
DED-related syndrome			0.834
1	16	19	
2	25	23	
≥ 3	22	21	

D: diopter, HA: hyaluronic acid, N: number, SE: spherical equivalent, UDVA: uncorrected distance visual acuity * denotes a significant difference after treatment

**Table 3:** Pretreatment parameters between the fair and poor outcome groups

Feature	Fair outcome group (N: 40)	Poor outcome group (N: 23)	P
Age (years, mean ± SD)	43.34±12.41	49.57±16.29	0.080
Sex (male: female)	12:28	3:21	0.218
Ocular disease			0.309
Retinal disease	1	2	
Glaucoma	0	1	
Uveitis	1	0	
Other	0	0	
Ocular surgery			0.007*
Retinal surgery	1	0	
Refractive surgery	0	5	
Other	0	0	
UDVA (LogMAR)	0.21±0.12	0.33±0.19	0.010*
Cycloplegia SE (D)	-2.77±2.01	-2.65±2.13	0.837
IOP	16.72±4.11	16.34±3.87	0.715
NITBUT	7.92±2.84	5.12±3.66	0.001*
Eyelid closure rate	95.34±8.27	92.82±8.96	0.197
Lipid thickness			0.503
Grade A-C	17	9	
Grade D-E	23	14	
TMH	0.33±0.19	0.26±0.20	0.181
Meibomian gland loss rate	25.27±17.45	37.47±16.35	0.006*
Schirmer II test	11.78±2.64	8.92±3.09	0.002*
Ocular surface stain			0.293
Grade 0-3	30	15	
Grade 4-6	10	8	
DED-related syndrome			0.828
1	11	5	
2	16	9	
≥ 3	13	9	

D: diopter, DED: dry eye disease, N: number, SD: standard deviation, SE: spherical equivalent, NITBUT: non-invasive tear break-up time, TMH: tear meniscus height, UDVA: uncorrected distance visual acuity * denotes a significant difference between groups

**Table 4:** Correlations between pretreatment parameters and poor dry eye disease symptom improvement

Factor	aOR	95% CI		P
		Lower	Upper	
Old age	1.231	0.956	1.428	0.115
Female sex	1.407	1.026	1.663	0.036*
Previous refractive surgery	1.683	1.254	2.130	0.001*
Low NITBUT	1.352	0.987	1.707	0.061
Low eyelid closure rate	1.002	0.876	1.138	0.484
Low lipid thickness	0.996	0.953	1.124	0.311
Low TMH	1.095	0.924	1.398	0.175
High meibomian gland loss rate	1.295	0.971	1.594	0.082
Low Schirmer II test	1.119	0.962	1.421	0.137
High ocular surface stain	1.148	0.907	1.389	0.183
Multiple DED symptoms	1.273	0.998	1.602	0.055

aOR: adjusted odds ratio, CI: confidence interval, DED: dry eye disease, NITBUT: non-invasive tear break-up time, TMH: tear meniscus height * denotes a significant correlation with poor dry eye disease sign improvement

**Table 5:** Correlations between pretreatment parameters and poor dry eye disease symptom improvement

Factor	aOR	95% CI		P
		Lower	Upper	
Old age	1.459	1.084	1.723	0.004*
Female sex	1.381	0.924	1.611	0.104
Previous refractive surgery	1.298	0.997	1.546	0.057
Low NITBUT	1.743	1.228	2.002	0.001*
Low eyelid blink rate	1.024	0.912	1.132	0.553
Low lipid thickness	1.037	0.945	1.218	0.411
Low TMH	1.001	0.961	1.079	0.629
High meibomian gland loss rate	1.582	1.096	1.923	0.026*
Low Schirmer II test	1.276	0.973	1.475	0.167
High ocular surface stain	1.334	0.980	1.671	0.088
Multiple DED symptoms	1.218	0.979	1.522	0.100

aOR: adjusted odds ratio, CI: confidence interval, DED: dry eye disease, NITBUT: non-invasive tear break-up time, TMH: tear meniscus height * denotes a significant correlation with poor dry eye disease sign improvement

## Abbreviations

aOR: adjusted odds ratio
CI: 95% confidence interval
DED: dry eye disease
IOP: intraocular pressure
IPL: intense pulse light
N: number
SD: standard deviation
SE: spherical equivalent
NITBUT: non-invasive tear break-up time
TMH: tear meniscus height
UDVA: uncorrected distance visual acuity
D: diopter

## References

[1] Clayton JA (2018). Dry Eye. N Engl J Med.

[2] Cai Y, Wei J, Zhou J, Zou W (2022). Prevalence and Incidence of Dry Eye Disease in Asia: A Systematic Review and Meta-Analysis. Ophthalmic Res.

[3] Sheppard J, Shen Lee B, Periman LM (2023). Dry eye disease: identification and therapeutic strategies for primary care clinicians and clinical specialists. Ann Med.

[4] Szczotka-Flynn LB, Maguire MG, Ying GS, Lin MC, Bunya VY, Dana R (2019). Impact of Dry Eye on Visual Acuity and Contrast Sensitivity: Dry Eye Assessment and Management Study. Optom Vis Sci.

[5] Pflugfelder SC, Stern ME (2020). The cornea in keratoconjunctivitis sicca. Exp Eye Res.

[6] Messmer EM (2015). The pathophysiology, diagnosis, and treatment of dry eye disease. Dtsch Arztebl Int.

[7] Fogagnolo P, Giannaccare G, Mencucci R, Villani E, Orfeo V, Aragona P (2024). Effectiveness of a New Active Tear Substitute Containing 0.2% Hyaluronic Acid and 0.001% Hydrocortisone on Signs and Symptoms of Dry Eye Disease by Means of Low- and High-Tech Assessments. Ophthalmol Ther.

[8] Fogagnolo P, Romano D, De Ruvo V, Sabella P, Rossetti L (2022). Clinical Efficacy of an Eyedrop Containing Hyaluronic Acid and Ginkgo Biloba in the Management of Dry Eye Disease Induced by Cataract Surgery. J Ocul Pharmacol Ther.

[9] Cagini C, Di Lascio G, Torroni G, Mariniello M, Meschini G, Lupidi M (2021). Dry eye and inflammation of the ocular surface after cataract surgery: effectiveness of a tear film substitute based on trehalose/hyaluronic acid vs hyaluronic acid to resolve signs and symptoms. J Cataract Refract Surg.

[10] Srinivasan S, Garofalo R, Williams R (2023). Safe and Effective Management of Dry Eye Symptoms with Hydroxypropyl Guar and Hyaluronic Acid Dual-Polymer Lubricating Eye Drops: A Review of Preclinical and Clinical Studies. Clin Ophthalmol.

[11] Yusufoğlu E, Keser S (2024). The effect of sodium hyaluronate on dry eye and corneal epithelial thickness following cataract surgery. Int Ophthalmol.

[12] Kojima T, Nagata T, Kudo H, Müller-Lierheim WGK, van Setten GB, Dogru M (2020). The Effects of High Molecular Weight Hyaluronic Acid Eye Drop Application in Environmental Dry Eye Stress Model Mice. Int J Mol Sci.

[13] Rolando M, Villella E, Loreggian L, Marini S, Loretelli C, Fiorina P (2023). Long-Term Activity and Safety of a Low-Dose Hydrocortisone Tear Substitute in Patients with Dry Eye Disease. Curr Eye Res.

[14] Cheng AMS, Tighe S, Sheha H, Tseng SCG (2018). Adjunctive role of self-retained cryopreserved amniotic membrane in treating immune-related dry eye disease. Int Ophthalmol.

[15] Mittal R, Patel S, Galor A (2021). Alternative therapies for dry eye disease. Curr Opin Ophthalmol.

[16] Suwal A, Hao JL, Zhou DD, Liu XF, Suwal R, Lu CW (2020). Use of Intense Pulsed Light to Mitigate Meibomian Gland Dysfunction for Dry Eye Disease. Int J Med Sci.

[17] Tashbayev B, Yazdani M, Arita R, Fineide F, Utheim TP (2020). Intense pulsed light treatment in meibomian gland dysfunction: A concise review. Ocul Surf.

[18] Demolin L, Es-Safi M, Soyfoo MS, Motulsky E (2023). Intense Pulsed Light Therapy in the Treatment of Dry Eye Diseases: A Systematic Review and Meta-Analysis. J Clin Med.

[19] Qin G, Chen J, Li L, Zhang Q, Xu L, Yu S (2023). Efficacy of intense pulsed light therapy on signs and symptoms of dry eye disease: A meta-analysis and systematic review. Indian J Ophthalmol.

[20] Miao S, Yan R, Jia Y, Pan Z (2022). Effect of Intense Pulsed Light Therapy in Dry Eye Disease Caused by Meibomian Gland Dysfunction: A Systematic Review and Meta-Analysis. Eye Contact Lens.

[21] Dell SJ (2017). Intense pulsed light for evaporative dry eye disease. Clin Ophthalmol.

[22] Toyos R, Desai NR, Toyos M, Dell SJ (2022). Intense pulsed light improves signs and symptoms of dry eye disease due to meibomian gland dysfunction: A randomized controlled study. PLoS One.

[23] Yang L, Pazo EE, Zhang Q, Wu Y, Song Y, Qin G (2022). Treatment of contact lens related dry eye with intense pulsed light. Cont Lens Anterior Eye.

[24] Costa VP, da Silva RS, Ambrósio R Jr (2013). The need for artificial tears in glaucoma patients: a comparative, retrospective study. Arq Bras Oftalmol.

[25] Bron AJ, de Paiva CS, Chauhan SK, Bonini S, Gabison EE, Jain S (2017). TFOS DEWS II pathophysiology report. Ocul Surf.

[26] Baudouin C, Aragona P, Messmer EM, Tomlinson A, Calonge M, Boboridis KG (2013). Role of hyperosmolarity in the pathogenesis and management of dry eye disease: proceedings of the OCEAN group meeting. Ocul Surf.

[27] Roda M, Corazza I, Bacchi Reggiani ML, Pellegrini M, Taroni L, Giannaccare G (2020). Dry Eye Disease and Tear Cytokine Levels-A Meta-Analysis. Int J Mol Sci.

[28] Kumar NR, Praveen M, Narasimhan R, Khamar P, D'Souza S, Sinha-Roy A (2023). Tear biomarkers in dry eye disease: Progress in the last decade. Indian J Ophthalmol.

[29] Seen S, Tong L (2018). Dry eye disease and oxidative stress. Acta Ophthalmol.

[30] Bu J, Liu Y, Zhang R, Lin S, Zhuang J, Sun L (2024). Potential New Target for Dry Eye Disease-Oxidative Stress. Antioxidants (Basel).

[31] Chen HC, Yang SF, Lee CY, Hsueh YJ, Huang JY, Chang CK (2024). Differences in change of post-operative antioxidant levels between laser-assisted lenticule extraction and femtosecond laser in situ keratomileusis. J Cell Mol Med.

[32] Rolando M, Zierhut M (2001). The ocular surface and tear film and their dysfunction in dry eye disease. Surv Ophthalmol.

[33] Barabino S (2022). Is dry eye disease the same in young and old patients? A narrative review of the literature. BMC Ophthalmol.

[34] Negrini S, Emmi G, Greco M, Borro M, Sardanelli F, Murdaca G (2022). Sjögren's syndrome: a systemic autoimmune disease. Clin Exp Med.

[35] Sweeney DF, Millar TJ, Raju SR (2013). Tear film stability: a review. Exp Eye Res.

[36] Matossian C, McDonald M, Donaldson KE, Nichols KK, MacIver S, Gupta PK (2019). Dry Eye Disease: Consideration for Women's Health. J Womens Health (Larchmt).

[37] Jackson CJ, Gundersen KG, Tong L, Utheim TP (2022). Dry eye disease and proteomics. Ocul Surf.

[38] Mastropasqua L, Barboni P, Savini G, Aragona E, D'Aloisio R, Lanzini M Refractive surgery and dry eye. Eur J Ophthalmol. 2023: 11206721231176312.

[39] Toda I (2018). Dry Eye After LASIK. Invest Ophthalmol Vis Sci.

[40] Vázquez A, Martínez-Plaza E, Fernández I, Sobas EM, González-García MJ, Enríquez-de-Salamanca A (2022). Phenotypic characterization of patients developing chronic dry eye and pain after refractive surgery: A cross-sectional study. Ocul Surf.

[41] Periman LM, Perez VL, Saban DR, Lin MC, Neri P (2020). The Immunological Basis of Dry Eye Disease and Current Topical Treatment Options. J Ocul Pharmacol Ther.

[42] Mejía LF, Gil JC, Jaramillo M (2019). Intense pulsed light therapy: A promising complementary treatment for dry eye disease. Arch Soc Esp Oftalmol (Engl Ed).

[43] Yurttaser Ocak S, Karakus S, Ocak OB, Cakir A, Bolukbasi S, Erden B (2020). Intense pulse light therapy treatment for refractory dry eye disease due to meibomian gland dysfunction. Int Ophthalmol.

